# Perception, knowledge, and attitudes towards molar incisor hypomineralization among Spanish dentists: a cross-sectional study

**DOI:** 10.1186/s12903-020-01249-6

**Published:** 2020-09-18

**Authors:** Clara Serna-Muñoz, Yolanda Martínez-Beneyto, Amparo Pérez-Silva, Andrea Poza-Pascual, Francisco Javier Ibáñez-López, Antonio José Ortiz-Ruiz

**Affiliations:** 1 Department of Integrated Paediatric Dentistry, Faculty of Medicine-Dentistry, University of Murcia, Murcia, Spain; 2 Unit of Preventive and Community Dentistry, Department of Dermatology, Stomatology, Radiology and Physical Medicine, Faculty of Medicine-Dentistry, University of Murcia, Hospital Morales Meseguer, 2a planta, C/ Marqués de los Vélez, s/n., 30007 Murcia, Spain; 3 Department of Stomatology, Faculty of Medicine-Nursing, University of the Basque Country, Leioa, Spain; 4 Scientific and Technical Research Area, Statistical Service, University of Murcia, Murcia, Spain

**Keywords:** Molar incisor hypomineralization, Knowledge, Perception, General dental practitioners, Paediatric dentists

## Abstract

**Background:**

Molar incisor hypomineralization (MIH) is a growing health problem, and its treatment is a challenge. The purpose of the present study was to evaluate and compare the perceptions, knowledge, and clinical experiences of MIH in general dental practitioners (GDPs) and paediatric dentists (PDs) in Spain.

**Methods:**

All dentists belonging to the College of Dentists of the Region of Murcia, in the South-East of Spain, were invited to participate in a cross-sectional survey. They were asked to complete a two-part questionnaire including sociodemographic profiles and knowledge, experience, and perceptions of MIH. Data were analysed using Pearson’s chi-square test, Fisher’s exact test and Cramer’s V test.

**Results:**

The overall response rate was 18.6% (214/1147). Most respondents were aged 31–40 years (44.86%), with more than 15 years of professional experience (39.72%). They worked mainly in the private sector (84.58%) and were licensed in dentistry (74.30%): 95.45% of PDs had detected an increase in the incidence of MIH in recent years (*p* <  0.001). Only 23.80% of GDPs claimed to have made a training course on MIH. With respect to the aetiology, chronic medical conditions (*p* = 0.029) and environmental pollutants (*p* = 0.008) were the only factors that showed significant between-group differences. Durability (*p* = 0.009) and remineralization potential (*p* = 0.018) were the factors where there was a between-group difference in the choice of the restoration material. In the case of post-eruptive fractures and opacities, the preferred material for both groups was resin-modified glass ionomer (RMGIC). However, in incisor lesions, composite was the material of choice for both groups, with significant differences (*p* = 0.032) in the use of glass ionomer. Most respondents expressed a need for continuing education on MIH.

**Conclusion:**

Spanish dentists perceived an increase in the incidence of MIH. The material of choice was RMGIC for non-aesthetic sectors and composite for incisors. Dentists believe it is difficult or very difficult to manage MIH, since the long-term success of restorations of MIH lesions is compromised because resin adhesion is not good. Both GDPs and PDs believe they need more training on the aetiology, diagnosis, and treatment of MIH.

## Background

The term molar incisor hypomineralization (MIH) was described by Weerheijm, et al. in 2001 and adopted by the international dental community due to a consensus at the Congress of the European Academy of Paediatric Dentistry in Athens in 2003 [1]. MIH is defined as a qualitative enamel developmental defect of systemic origin that affects one or more first permanent molars with or without the involvement of permanent incisors [1]. When it appears in primary teething it is called hypomineralized second primary molar (HSPM), and predominantly affects the second molars and canines and is regarded as a predictive factor for MIH in the permanent teeth [2].

Historically, in the dental literature, a wide variety of terminology and definitions for enamel defects in hypomineralized molars, with or without post-eruptive enamel fractures have been used: enamel opacity not caused by fluoride, internal enamel hypoplasia, non-endemic enamel speckling, opaque stains, idiopathic enamel opacities, and enamel opacity. Some terms simply describe the pathology, while others bear the name of the causal agent [3]. Despite the many reports on its aetiology, the causal factors of MIH remain unclear [4].

Clinically, the form of presentation and the severity of MIH-affected teeth may be asymmetrical in the same patient and vary from mild opacities to severe post-eruptive breakdown that may affect from one to four first permanent molars [5]. MIH may be difficult to diagnose, and it may be confused with other conditions such as enamel hypoplasia, fluorosis and amelogenesis imperfecta. However, in enamel hypoplasia, the lesion consists of a local reduction of the thickness of the enamel, with regular, smooth and rounded borders while, in enamel fractures with MIH, the borders are irregular and anfractuous; the enamel opacities observed in fluorosis are diffuse and symmetrical, in contrast to the well-demarcated lesions of the enamel in MIH; lesions due to amelogenesis affect all the teeth, as this is a hereditary, genetic disorder, while lesions due to MIH are asymmetric and are located in the first permanent molars and incisors. In addition, the diagnosis may be complicated by secondary cavity lesions due to their rapid formation and progression in a highly-porous substrate [6].

The global recorded prevalence of MIH ranges from 2.4 to 40% and differs between countries [7, 8]. There are a limited number of studies on the prevalence of MIH in Spain, where the prevalence varies from 7.94% [9] to 11.1% [10], 17.8% [11] and 21.8% [12].

The high prevalence and incidence of MIH, the poor quality of life of paediatric patients and the difficult clinical management, have led to numerous studies on the perception, diagnosis and management of MIH through surveys of dentists, both general dental practitioners (GDPs) and paediatric dentists (PDs) [13–22]. In Spain, despite the high prevalence described in some areas, no studies have been conducted on how dentists act in the face of MIH. The objective of this study was to evaluate perceptions and knowledge about the diagnosis and management strategies of MIH of GDPs and PDs in the Region of Murcia, in the south east of Spain.

## Methods

### Sample and procedures

The study was approved by the Bioethics Committee of Murcia University (Reference Number: 2255/2019). Google Survey software was used to develop the survey that was subsequently emailed to all members of College of Dentists of Murcia (*n* = 1147) in March 2019. The email explained the study, stated that participation was anonymous and voluntary, and enclosed a link to accede to the survey without signing in to Google. The study researchers had no access to the personal data of participants. Participants were asked to complete the online questionnaire on their own time. A reminder email was sent 2 weeks after initial distribution. The survey was online for 1 month.

A pilot version of the questionnaire was tested by six teachers and six postgraduate students of the master’s degree in Integrated Paediatric Dentistry, University of Murcia, to ensure the questions had been correctly prepared, were easily understandable and did not entail a prolonged response time.

### Survey instrument (Supplementary file 1)

The questionnaire was divided into two main sections. The first section covered demographics (age group, years of practice, occupational sector, qualification), educational background (main area of work), perception (changes in the incidence of MIH lesions in recent years), clinical appearance (severity of MIH lesions, similar lesions in the second temporary molar), prevalence (how often do they see MIH lesions, how many patients present them), participants’ attitudes, knowledge (aetiology) and practice in MIH management and, finally, restorative options in MIH management (types of restoration material and factors that influence the choice).

**Fig. 1:**
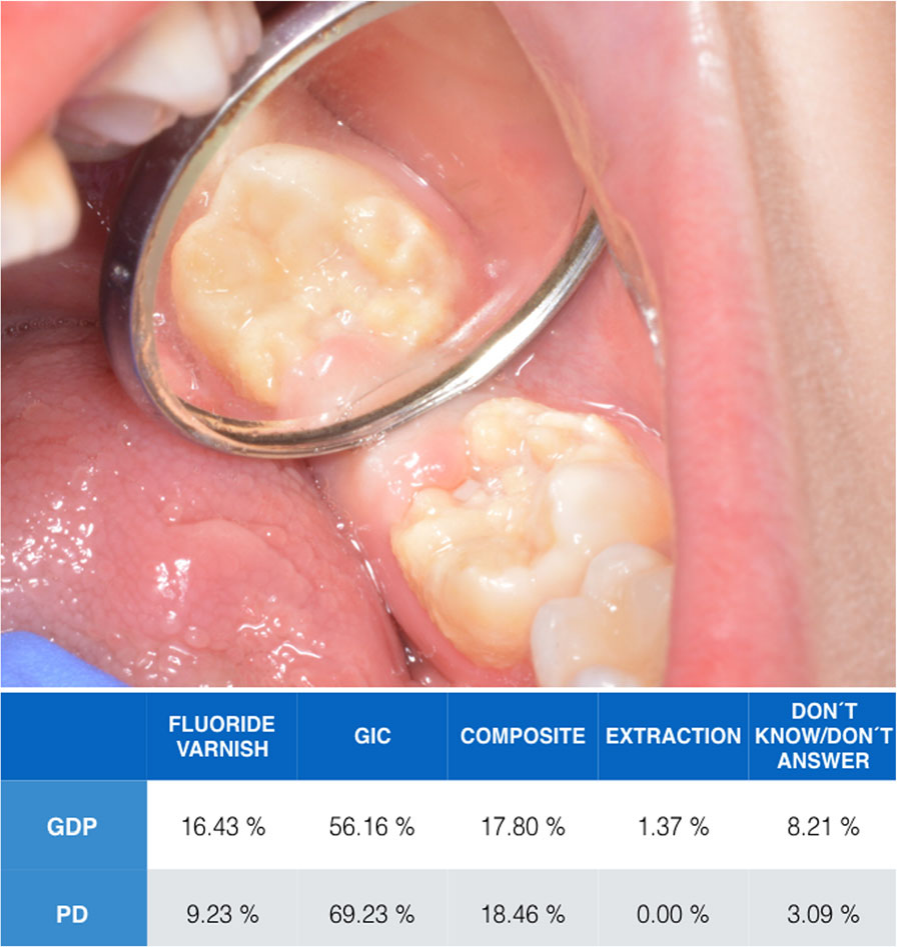
In clinical case 1, dentists were asked which treatment they would prefer for a semi-erupted primary molar with moderate MIH, post-eruptive fracture and sensitivity in the tooth in a seven-year-old patient. The options were: (1) Fluoride varnish, (2) Restoration with glass ionomer cement (GIC) (3) Restoration with resin composite (4) Extraction of the tooth (5) I am not sure of the best option

**Fig. 2:**
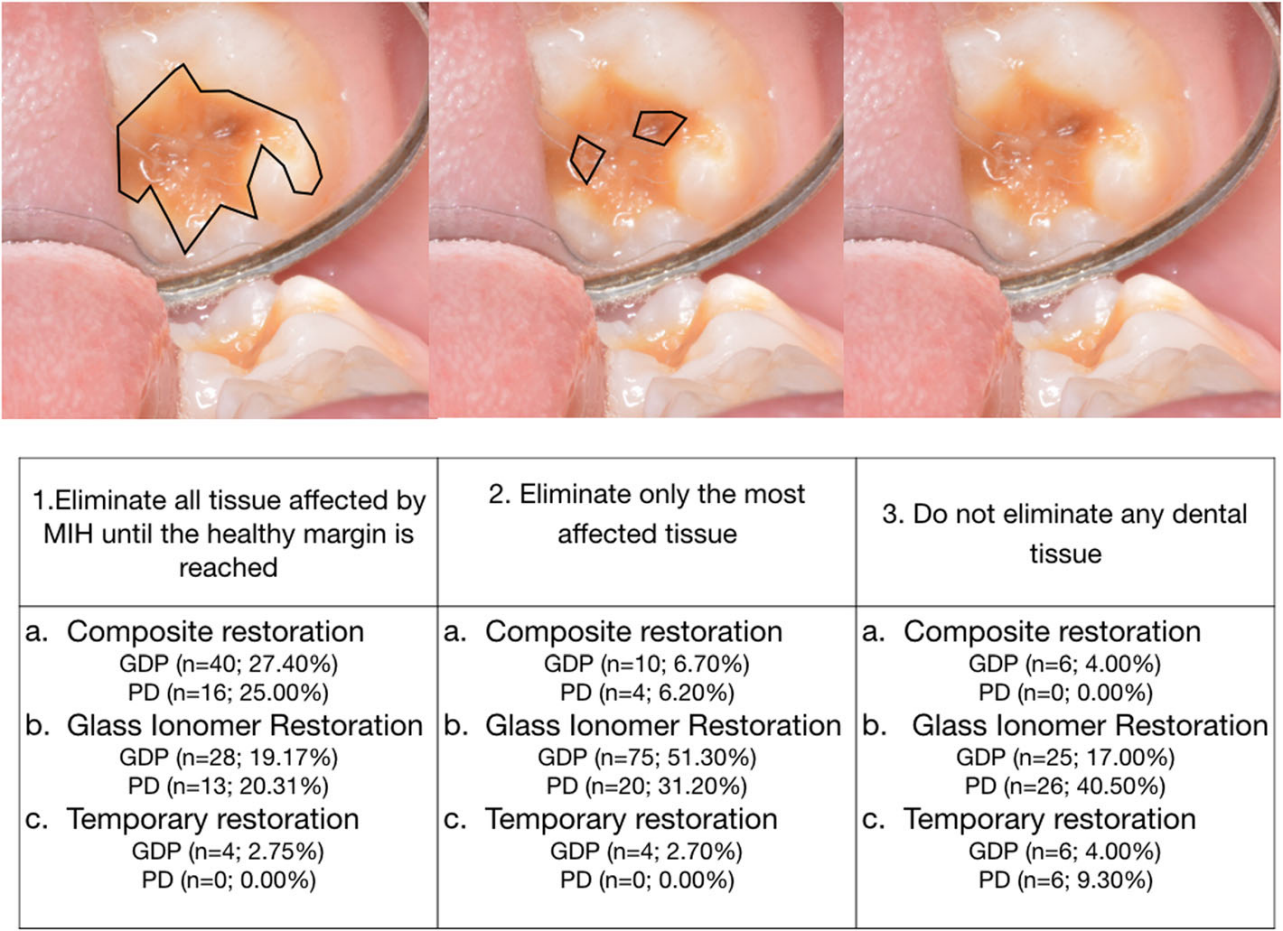
In clinical case 2, dentists were asked about the best treatment for a delimited brown opacity without post-eruptive enamel fracture. The options were: (1A) Eliminate all tissue affected by MIH and restore with resin composite; (1B) Eliminate all affected tissue and restore with glass ionomer cement (GIC); (1C) Eliminate all affected tissue and make a temporary restoration; (2A) Eliminate only the most affected tissue and restore with composite; (2B) Eliminate only the most affected tissue and restore with glass ionomer; (2C) Eliminate only the most affected tissue and make a temporary restoration; (3A) Do not eliminate any dental tissue and restore with composite;(3B) Do not eliminate any dental tissue and restore with glass ionomer (GIC); (3C) Do not eliminate any dental tissue and make a temporary restoration

In the second part of the questionnaire, two clinical situations with illustrative photographs and a written case description were suggested to dentists. In clinical case 1 (Fig. 1), dentists were asked which treatment they would prefer for a semi-erupted first permanent molar with moderate MIH, post-eruptive fracture and sensitivity in the tooth in a seven-year-old patient. The options were: fluoride varnish, restoration with GIC, restoration with composite, extraction and “I am not sure of the best option”. In clinical case 2 (Fig. 2), dentists were asked about the best treatment for a delimited brown opacity without post-eruptive enamel fracture. The options were: eliminate all tissue affected by MIH and restore, eliminate only the most affected tissue and restore, and do not eliminate any dental tissue and restore. The options for restorative material were resin composite, GIC and temporary restoration.

The questions in the first section of the questionnaire, where it says “tick one option”, and the first clinical case have only one answer. The second clinical case, and the questions where it says “choose the corresponding answers”, are multiple choice, so the number of responses could be greater than the number of respondents.

### Data analysis

Study data were processed and analysed using the R statistical package. A simple frequency distribution was made. Independent variables (sociodemographic variables: Table 1) and dependent variables (remaining survey questions: Tables 2, 3 and 4) were tabulated for GDPs and PDs. To identify differences in dependent variables between GDPs and PDs, Pearson’s chi-square test was applied in contrasts where the required assumptions were met, and Fisher’s exact test in which they were not (*p*-value < 0.05 and significance level 0.05). Cramer’s test was used to determine whether the relationship was strong or weak.

**Table 1:** Demographic characteristics of study participants

Characteristics	Total n (%)	GDPs n (%)	PDs n (%)
**Age group**	214 (100)	148 (100)	66 (100)
< 30	56 (26.17)	37 (25.00)	19 (28.79)
31–40	96 (44.86)	67 (45.27)	29 (43.94)
41–50	33 (15.42)	23 (15.54)	10 (15.15)
> 50	29 (13.55)	21 (14.19)	8 (12.12)
**Years of practice**	214 (100)	148 (100)	66 (100)
< 5	47 (21.96)	35 (23.65)	12 (18.18)
6–10	37 (17.29)	24 (16.22)	13 (19.70)
11–15	45 (21.03)	33 (22.30)	12 (18.18)
> 15	85 (39.72)	56 (37.84)	29 (43.94)
**Work Sector**	214 (100)	148 (100)	66 (100)
Public sector	7 (3.27)	3 (2.03)	4 (6.05)
Private Sector	181 (84.58)	130 (87.83)	51 (77.26)
Combined	26 (12.15)	15 (10.13)	11 (16.67)
**Qualification (degree level)**			
Stomatologist	23 (10.75)	18 (12.162)	5 (7.57)
Dentistry Licenciated (up to 2010)	159 (74.30)	104 (70.27)	55 (83.33)
Dentistry Graduated (later than 2010)	32 (14.95)	26 (17.57)	6 (9.08)

*GDPs* General dental practitioners, *PDs* Pediatric dentists

**Table 2:** MIH perception, clinical appearance and prevalence according to study participants

Question	Total n (%)	GDPs n (%)	PDs n (%)	***P***-Value
**How often do you notice hypomineralized teeth in your practice?**	214 (100)	148 (100)	66 (100)	**< 0.001**
Weekly	88 (41.12)	40 (27.02)	48 (72.73)	
Monthly	104 (48.59)	88 (59.46)	16 (24.24)	
Annually	22 (10.28)	20 (13.50)	2 (3.03)	
**Approximately what percentage of your patients present this defect?**	214 (100)	148 (100)	66 (100)	**< 0.001**
< 10%	104 (48.59)	88 (59.45)	16 (24.24)	
10–25%	87 (40.65)	54 (36.48)	33 (50.00)	
> 25%	23 (10.74)	6 (4.05)	17 (25.76)	
**Do you perceive that the incidence of MIH has increased in recent years?**	214 (100)	148 (100)	66 (100)	**< 0.001**
No	41 (19.15)	38 (25.67)	3 (4.54)	
Yes	173 (80.84)	110 (74.32)	63 (95.45)	
**What do you most frequently notice in your practice?**	214 (100)	148 (100)	66 (100)	0.375
White demarcated opacities	78 (36.45)	58 (39.19)	20 (30.30)	
Yellow/brown demarcated opacities	129 (60.28)	86 (58.11)	43 (65.16)	
Post-eruptive enamel breakdown	7 (3.27)	4 (2.7)	3 (4.54)	
**How frequently do you notice this defect in the second primary molar?**	210 (100)	144 (100)	66 (100)	0.516
More often	9 (4.28)	5 (3.47)	4 (6.06)	
Equally as often	15 (17.14)	12 (8.33)	3 (4.54)	
Less often	186 (88.57)	127 (88.19)	59 (89.39)	

*GDPs* General dental practitioners, *PDs* Pediatric dentists

**Table 3:** MIH management considerations, source of information, and clinical training demand according to study participants

Question	Total n (%)	GDPs n (%)	PDs n (%)	***P***-Value
**Which factors do you think are involved in the etiology of MIH?**^**a**^	**827 (100)**	**535 (100)**	**292 (100)**	
Genetic factors	107 (12.93)	77 (14.39)	30 (10.27)	0.459
Acute medical condition that affects the mother during pregnancy	110 (13.30)	74 (13.83)	36 (12.32)	0.641
Acute medical condition that affects the child involved	93 (11.24)	61 (11.40)	32 (10.95)	0.401
Antibiotics/medications taken by the mother during pregnancy	90 (10.88)	56 (10.46)	34 (11.64)	0.085
Antibiotics/medications taken by the child involved	115 (13.90)	73 (13.64)	42 (14.72)	0.073
Chronic medical condition that affects the mother during pregnancy	75 (9.06)	47 (8.78)	28 (9.58)	0.175
Chronic medical condition that affects the child involved	76 (9.18)	45 (8.41)	31 (10.61)	**0.029**
Environmental contaminants	96 (11.60)	57 (10.65)	39 (13.35)	**0.008**
Fluoride exposure	65 (7.85)	45 (8.41)	20 (6.85)	1
**Do you think the management of MIH is a challenge?**	**213 (100)**	**148 (100)**	**65 (100)**	0.837
Yes, very difficult	72 (33.80)	52 (35.13)	20 (30.76)	
Yes, somewhat difficult	127 (59.62)	86 (58.10)	41 (63.07)	
No	14 (6.57)	10 (6.76)	4 (6.15)	
**Which are the biggest difficulties?**^**a**^	**649 (100)**	**439 (100)**	**210 (100)**	
Diagnosis	42 (6.47)	29 (6.60)	13 (6.19)	1
Esthetics	66 (10.17)	42 (9.57)	24 (11.42)	0.385
Long-term success of restoration	173 (26.65)	120 (27.33)	53 (25.23)	0.657
Correct determination of restoration margins	124 (19.10)	89 (20.27)	35 (16.66)	0.266
Achieving correct local anesthetic	72 (11.09)	42 (9.57)	30 (14.28)	**0.031**
Providing correct restoration	144 (22.18)	100 (22.77)	44 (20.95)	0.765
Other	28 (4.31)	17 (3.87)	11 (5.29)	0.464
**Do you receive any information on MIH?**	**213 (100)**	**147 (100)**	**66 (100)**	**< 0.001**
Yes	74 (34.74)	35 (23.80)	39 (59.09)	
No	139 (65.25)	112 (76.19)	27 (40.90)	
**Where do you obtain the information**	**194 (100)**	**130 (100)**	**61 (100)**	**0.005**
Journals	34 (17.52)	24 (18.46)	10 (16.39)	
Continuing education	54 (27.83)	27 (20.77)	27 (44.26)	
Brochures	4 (2.06)	4 (13.07)	0 (0)	
Internet	58 (29.89)	47 (36.15)	11 (18.03)	
Books	10 (5.15)	5 (3.84)	5 (8.19)	
Others	31 (15.98)	23 (17.69)	8 (13.11)	
**Where do you think more information is necessary?**	**212 (100)**	**146 (100)**	**66 (100)**	**0.023**
Etiology	19 (8.96)	8 (5.47)	11 (16.66)	
Diagnosis	1 (0.47)	0 (0.00)	1 (1.51)	
Treatment	69 (32.54)	51 (34.93)	18 (27.27)	
All	123 (58.01)	87 (59.59)	36 (54.54)	

*GDPs* General dental practitioners, *PDs* Pediatric dentists.

^a^ These questions are multiple choice, so the number of responses could be greater than the number of respondents (*n* = 214)

**Table 4:** Restorative management options for molar incisor hypomineralization (MIH)

Question	Total n (%)	GDPs n (%)	PDs n (%)	***P***-Value
**Factors in the choice of material**^**a**^	**577 (100)**	**392 (100)**	**185 (100)**	
Adhesion	137 (23.74)	96 (24.48)	41 (22.16)	0.817
Durability	124 (21.49)	95 (24.23)	29 (15.67)	**0.009**
Experience	30 (5.20)	18 (4.59)	12 (6.48)	0.338
Remineralization potential	146 (25.30)	93 (23.72)	53 (13.52)	**0.018**
Patient/parent preferences	8 (1.38)	6 (1.53)	2 (0.51)	1
Sensitivity	84 (14.55)	53 (13.52)	31 (7.91)	0.175
Research findings	48 (8.32)	31 (7.91)	17 (4.33)	0.547
**Material of choice for post-eruptive fractures**^**a**^	**387 (100)**	**262 (100)**	**125 (100)**	
Compomer	13 (3.35)	10 (3.81)	3 (2.4)	0.758
Composite resin	88 (22.74)	59 (22.51)	29 (23.2)	0.674
Flowable composite resin	18 (4.65)	15 (5.72)	3 (2.40)	0.285
Stainless steel crown	38 (9.82)	26 (9.92)	12 (9.60)	1
Silver diamine fluoride	6 (1.55)	4 (1.53)	2 (1.60)	1
Cast restoration	23 (5.94)	16 (6.11)	7 (5.60)	1
GIC	55 (14.21)	32 (12.21)	23 (18.40)	**0.048**
RMGIC	139 (35.91)	95 (36.25)	44 (35.20)	0.831
Others	7 (1.81)	5 (1.90)	2 (1.60)	1
**Material of choice for opacities**^**a**^	**316 (100)**	**216 (100)**	**100 (100)**	
Amalgam	4 (1.26)	4 (1.85)	0 (0.00)	0.315
Compomer	18 (5.69)	15 (6.94)	3 (3.00)	0.284
Composite resin	87 (27.53)	61 (28.24)	26 (26.00)	0.966
Flowable composite resin	25 (7.91)	17 (7.87)	8 (8.00)	1
Stainless steel crowns	6 (1.89)	5 (2.31)	1 (1.00)	0.669
Silver diamine fluoride	18 (5.69)	11 (5.09)	7 (7.00)	0.597
GIC	42 (13.29)	25 (11.57)	17 (17.00)	0.173
RMGIC	109 (34.49)	72 (33.33)	37 (37.00)	0.348
Others	7 (2.21)	6 (2.77)	1 (1.00)	0.678
**Material of choice for hypomineralized incisors**^**a**^	**318 (100)**	**209 (100)**	**109 (100)**	
Compomer	12 (3.77)	8 (3.82)	4 (3.66)	1
Composite resin	122 (38.36)	86 (41.14)	36 (33.02)	0.794
Flowable composite resin	40 (12.57)	27 (12.91)	13 (11.95)	0.924
Stainless steel crowns	1 (0.31)	1 (0.47)	0 (0.00)	1
Silver diamine fluoride	3 (0.94)	2 (0.95)	1 (0.91)	1
Resin infiltration	52 (16.35)	35 (16.74)	17 (15.59)	0.841
GIC	15 (4.71)	7 (3.34)	8 (7.33)	0.077
RMGIC	62 (19.49)	36 (17.22)	26 (23.85)	**0.032**
Other	11 (3.45)	7 (3.34)	4 (3.66)	0.739

*GDPs* General dental practitioners, *PDs* Pediatric dentists, *GIC* Glass ionomer cement, *RMGIC* Resin-modified glass ionomer cement

^a^ These questions are multiple choice, so the number of responses could be greater than the number of respondents (*n* = 214)

## Results

Of the 1147 dentists invited to participate, 216 responded. Two surveys were eliminated because they were not completed correctly, resulting in a response rate of 18.66% (*n* = 214): 69.16% were GDPs or other specialties (*n* = 148), and 30.84% were PDs (*n* = 66).

Of the participants, 44.86% were aged 31–40 years, 39.72% had > 15 years of practical experience, 84.58% worked in the private sector, and 74.30% were licensed in Dentistry (Table 1).

The perception of GDPs and PDs about MIH is shown in Table 2: 59.46% of GDPs make diagnoses of MIH monthly, while 72.73% of PDs diagnose MIH weekly.

In terms of prevalence, 59.45% of GDPs found that < 10% of their patients had MIH, while 50.00% of PDs said that 10–25% of their patients had MIH. In addition, 95.45% of PDs had detected an increase in the incidence of MIH in recent years (*p* <  0.001).

Yellow-brown demarcated opacities were the most common clinical forms detected, both by PDs (65.16%) and GDPs (58.11%) and were most often diagnosed in the permanent teething.

The knowledge of respondents regarding the aetiology of MIH is shown in Table 3. Many factors were mentioned, but chronic medical conditions affecting children (*p* = 0.029) and environmental pollutants (*p* = 0.008) were the only factors that showed significant differences between the two groups.

As for the difficulty of managing MIH, the most frequent response was that it is considered a challenge and that GPDs (76.19%) had not received any information on this (*p* < 0.001). PDs stated that the information they obtain on MIH basically comes from face-to-face continuing education (44.26%), while the Internet was the source of choice for GDPs (36.15%) (*p* = 0.005), with widespread demand for information on the aetiology, diagnosis and treatment of MIH.

The results on restorative treatments in MIH are shown in Table 4. Significant differences between GDPs and PDs in the choice of material characteristics were identified, such as durability (*p* = 0.009) and remineralization potential (*p* = 0.018). As for the material of choice in cases of post-eruptive fractures, RMGIC was the most widely used by both groups. However, there were significant differences in the use of glass ionomer cement (GIC) (*p* = 0.048) between GDPs (12.21%) and PDs (18.40%). No significant differences were found in the materials used to restore opacity, with RMGIC again being the first choice in both groups. However, in the case of the treatment of lesions in the incisors, composite was the material of choice in both groups, with significant differences (*p* = 0.032) in the use of RMGIC between GDPs and PDs.

In the second part of the questionnaire, in the first clinical case (Fig. 1), the material of choice for most respondents was GIC in both groups (GDPs: 56.1% vs. PD: 69.1%). In the second clinical case (Fig. 2), 51% of GDPs supported removal of only the most affected tissue and restoration with GIC. However, a more conservative attitude was observed among PDs (40.5%), who stated they would not remove any enamel and would restore with GIC.

## Discussion

Despite the high prevalence and increased incidence of MIH in Spanish paediatric patients, this is the first study to provide information on the perception and knowledge of the aetiology and diagnosis of MIH and patient management strategies of Spanish GDPs and PDs.

We used an online survey to avoid the low response rate obtained in postal surveys [23]. The response rate was 18.6%, despite a reminder sent at 2 weeks. Although the response was low, it was similar to that recorded in similar studies in other countries [8, 13, 16, 19, 21] and in various dental studies [24, 25]. The response rate in surveys of health care workers is falling, despite the use of new technologies. However, surveys continue to be an important source of information on the knowledge, attitudes, opinions, and practices associated with new or controversial topics [26]. The response rate has often been regarded as a measure of the quality of the work, but there is no scientifically accepted minimum response rate. Non-response bias, meaning respondents do not represent the target population, is more of a problem in surveys in the general population than in those in specific groups such as physicians [27] or, in our case, dentists.

In Spain, dentistry is mostly private, which is reflected by the responders (84.58% private), unlike countries such as Norway [14] or Australia and Chile [19] where most dentists are public. We found that 69.16% of participants were GDPs and 30.84% PDs. PD training in Spain is not specialized, as in most European Union countries, but is a postgraduate master. In both groups of dentists, the majority (74.30%) of practitioners were licensed in Dentistry, with a mean age of 31–40 years (44.86%) and with > 15 years of professional experience (39.72%). The professional profiles found in other studies vary in age and years of experience, with the study conducted in Hong Kong having the oldest professionals and the greatest professional experience [8].

We found that PDs had twice the perception of patients with MIH lesions compared with GDPs, a situation reflected in countries such as Iraq [15], Malaysia [22], Australia-New Zealand [18], Saudi Arabia [17], China [8] and the UK [20], where the prevalence of MIH is similar to Spain. Both in Spain and in other countries [15, 17, 18], the general perception of dentists is that there is an increase in the incidence of MIH, although in our case the perception is significantly higher in PDs than in GDPs. Thus, 59.45% of GDPs responded that the prevalence of MIH patients is < 10%. GDPs from countries such as the USA [21] India [16] and China [8] estimated the prevalence at < 5%. These results are closely related to the training of dentists and their diagnostic ability: 59.09% of PDs claimed to have training in MIH compared with 23.80% of GDPs. In addition, training was received in continuous education courses, compared with the online self-training described by GDPs. In other countries, the training of PDs in MIH shows similar results, although GDPs had less training in MIH (7.0–8.8%) [16, 28]. Despite these results, both PDs and GDPs in Spain require ongoing training courses on MIH [8, 15, 16, 19].

The most recognized MIH lesion in both study groups was yellow/brown lesions, as it was in other countries [13, 15–18]. This may be because white-cream lesions may be mistaken for other lesions, such as fluorosis or white spot cavities [20, 29]. The percentage of post-eruptive enamel fractures was low, possibly because they may be confused with extensive cavity lesions, with atypical restorations typical of this pathology, since the enamel breaks quickly after rupture [15, 30], or with enamel hypoplasia, although in this case the borders of the lesion are not as irregular as in MIH [20].

In 2012, hypomineralization of the primary teeth was described, mainly in the second primary molars (HSPM). This is known to be associated with an increased risk of hypomineralization in the permanent molars, although the absence of HSPM does not exclude future MIH [2]. Most of our respondents report detecting HSPM less frequently, with no differences between the two groups, as is the case in studies in the USA [21], Kuwait [13], Saudi Arabia [17] and Australia-Chile [19], even though PDs have greater access to paediatric populations, where the diagnosis should be more common.

In general, and as in other studies [8, 15–18, 21], dentists’ responses reflect the hypothesis that the aetiology may be multifactorial, with a diversity of responses. Most studies, when describing etiological factors, attribute MIH primarily to chronic and acute medical conditioners affecting the mother and child [8, 15, 16, 19]. In our study, 42.80% of dentists attributed the aetiology to these factors, lower than the 80–100% found in other studies [8, 18]. The second cause, in our study, was the consumption of antibiotics by the child or mother during pregnancy (24.78%), figures similar to the Iran study [15] but below the studies in Hong Kong [8] and Australia-New Zealand [18]. Environmental pollutants were considered causal agents by 11.6%, with a different perception between GDPs and DPs.

A significant percentage of both GDPs and PDs responded that they found the management of MIH “somewhat difficult”. This is because these patients have increased anxiety [31] and tooth hypersensitivity, even after local anaesthesia. In fact, anaesthesia is one of the procedures that mark significant differences between dentists, with GDPs finding it more difficult to achieve good anaesthesia than PDs.

Achieving correct restoration and long-term success is what worries dentists the most (48.84%). It is known that etching with orthophosphoric acid creates faulty etched patterns [32], that resin penetration is defective and the adhesion force of the composite resins to the enamel affected by MIH is low [33], and that there is a high failure rate of this type of material in molars with MIH [34]; in fact, the second most relevant factor in the choice of material by our dentists is material adhesion (23.74%).

There are many reported treatment options for the restoration of teeth with MIH lesions: fluoride and/or CPP-ACP remineralization systems, silver diamine fluoride, pit and fissure sealants, resin infiltrations, conventional and modified glass ionomers with resin, resin composite, amalgam, preformed crowns, and even extractions, always depending on the severity of the lesion [28].

The potential for remineralization of material in restoration is the most relevant factor in the choice of materials (25.3%), significantly worrying GDPs more than PDs. In fact, the most commonly used material to restore post-eruptive fractures is RMGIC, followed by composite and both are used equally by GDPs and PDs. GIC is the third material of choice and is used proportionally more by PDs than GDPs. This may be because they treat younger children and use it as filling material in atraumatic restorative treatments or for interim restorations. Durability, which is one of the most relevant factors in material choice, is therefore significantly less decisive for PDs than for GDPs.

There are studies that show GIC (81%) was used more than RMGIC (44.3%), which is justified by the greater fluoride release [18]. However, a recent systematic review showed that the failure rate of restorative materials in the treatment of MIH is higher with the use of amalgams and glass ionomers, and the highest success rate is achieved with indirect restorations, preformed stainless steel crowns (SSC) and composite restorations [28]. In other studies, composite was the material of choice [8, 13, 17, 18], and was recommended by Lygidakis et al. [30] in moderate lesions. In our study, the number of SSCs was very low compared with other studies [8, 16, 18] in which it was the treatment of choice in fractures for most PDs. Some authors recommend them for moderate and severe MIH lesions instead of GIC and RMGIC [30].

With respect to enamel opacity, the materials chosen were the same in both groups; first RMGIC followed by composite. However, in incisor lesions, composite was the material of choice, due to aesthetic concerns, followed by RMGIC and resin infiltrations. PDs use significantly more RMGIC to restore incisor enamel lesions.

The adhesion, durability and potential for remineralization were also decisive in the choice of material by most dentists from other countries [8, 16, 18]. We left open the possibility of “other materials” where dentists could introduce other options used in combination with those defined in the survey. However, there were only 1.81–3.45% of responses. In contrast, in the Hong Kong study, 96.3% of PDs used fluoride varnishes and 64% pit fissure sealants [8].

In clinical case 1 (Fig. 1), where a post-eruptive enamel fracture was presented in a semi-erupted tooth, the material selected by both groups for treatment was GIC, followed by composite, similar to the results of the Norwegian study [14]. Difficult moisture control in a semi-erupted molar and fluoride release were the main reasons for choosing GIC. The limited mechanical properties of GIC mean it should be considered an interim therapeutic restoration and must be replaced by another, definitive material (composite or preformed crowns) when eruption is complete [31].

In clinical case 2, the preferred option for GDPs was to remove the tissue seemingly most affected and restore with glass ionomer, compared with PDs whose option was not to remove any dental tissue and use glass ionomer to restore. This shows a trend towards less invasive treatment by PDs, as described by other reports [14].

The study had some limitations. First, the response rate was low, although this is characteristic of online surveys. Secondly, it was aimed at dentists from one Spanish region. Therefore, extrapolation of the results to the rest of Spain, should be made with caution, as sociodemographic characteristics may vary. However, the survey may serve as a starting point for the introduction of Spanish guidelines or protocols on the correct care of children with MIH.

## Conclusion

Spanish dentists perceive that the incidence of MIH has increased in recent years. They believe it is difficult or very difficult to manage MIH, since the long-term success of restorations of MIH lesions is compromised because resin adhesion is not good. They use RMGICs more frequently, taking advantage of their remineralizing potential, except in the incisors, where they use composites. Both GDPs and PDs think they need more training on the aetiology, diagnosis, and treatment of MIH. The introduction of national guidelines that serve as a reference manual for all continuing education courses would improve the management of MIH.

## Supplementary information

**Additional file 1.**

## Data Availability

The datasets used for the current study are available from the corresponding author on reasonable request.

## Abbreviations

MIH: Molar incisor hypomineralization
GDPs: General dental practitioners
PDs: Paediatric dentists
RMGIC: Resin-modified glass ionomer
HSPM: Hypomineralized second primary molar
GIC: Glass ionomer cement
UK: United Kingdom
SSC: Stainless steel crowns
