# Supplementary material for: Perception, knowledge, and attitudes towards molar incisor hypomineralization among Spanish dentists: a cross-sectional study
Source: BMC Oral Health. 2020 Sep 18;20:260. doi: 10.1186/s12903-020-01249-6 (PMC7501634; doi:10.1186/s12903-020-01249-6)
Supplement: Supplementary file 1 — Additional file 1. [file 12903_2020_1249_MOESM1_ESM.pdf]

Perception, knowledge and attitudes towards molar incisor hypomineralization among spanish dentists: a cross-sectional study.

Clara Serna-Muñoz, Yolanda Martínez-Beneyto, Amparo Pérez-Silva, Andrea Poza-Pascual, Francisco Javier Ibáñez-López, Antonio José Ortiz-Ruiz.

1. Age group

Tick one option

- ○ < 30
- ○ 31-40
- ○ 41-50
- ○ >51

2. Years of practice

Tick one option

- ○ <5
- ○ 6-10
- ○ 11-15
- ○ >15

3. Sector of work

Tick one option

- ○ Public sector
- ○ Private sector
- ○ Both

4. Qualification

Tick one option

- ○ Stomatologist
- ○ Licensed
- ○ Graduate

5. What is your main area of work?

Choose the corresponding answers

- ☐ Pediatric dentistry
- ☐ General dentistry
- ☐ Surgery
- ☐ Orthodontics
- ☐ Root canal
- ☐ Aesthetic
- ☐ Others

6. Do you usually see this type of lesions in your practice?

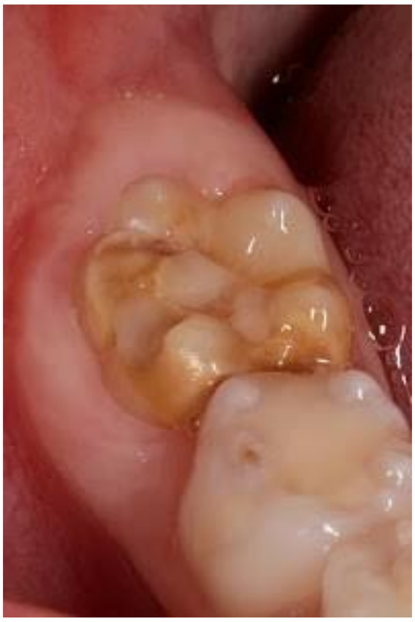

Tick one option

- ○ Yes
- ○ No

7. Do you usually see this type of lesions in your practice?

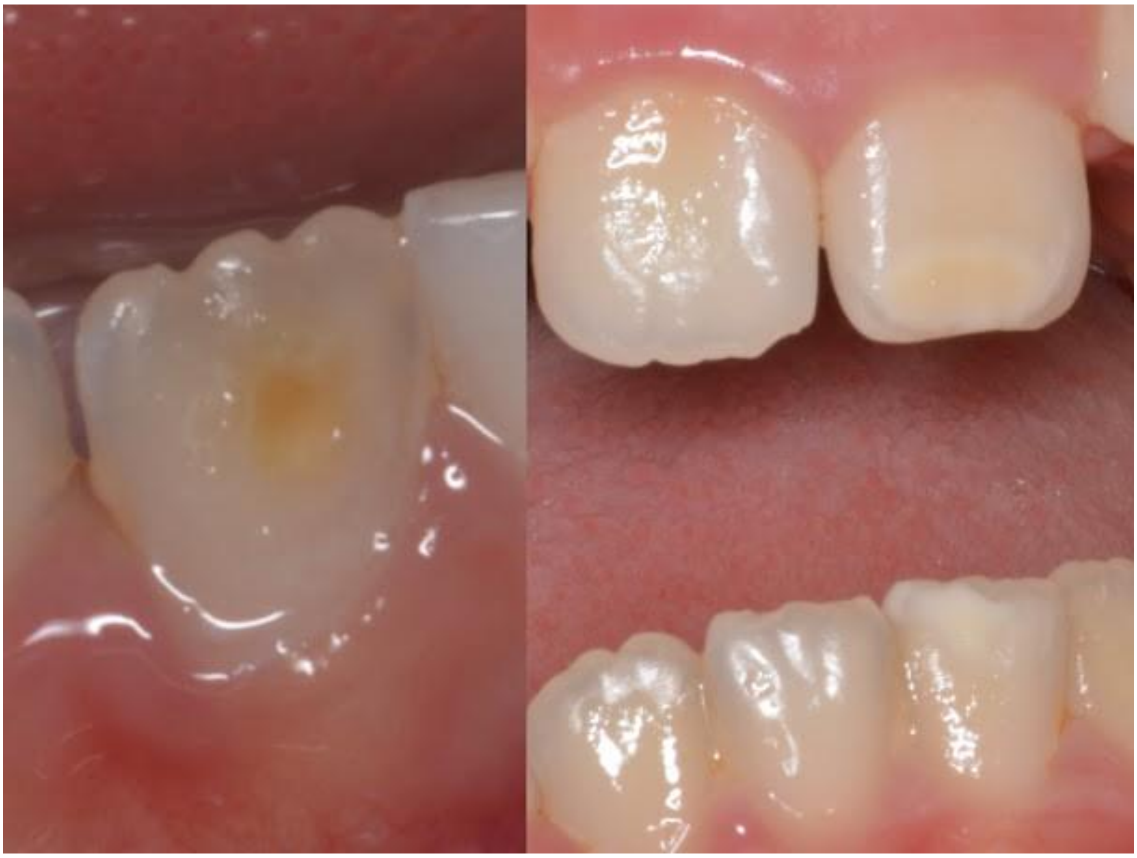

Tick one option

- ○ Yes
- ○ No

8. How often do you see them?

Tick one option

- ○ Weekly
- ○ Monthly
- ○ Annually

9. Approximately how many of your patients present these lesions?

Tick one option

- ○ <10%
- ○ 10-25%
- ○ >25%

10. In your practice, have you noted that the incidence of MIH has risen in recent years?

Tick one option

- ○ Yes
- ○ No

11. With respect to the severity of the defect. Which of the following lesions do you see most often in clinical practice?

Tick one option

- ○ White-cream opacity
- ○ Yellow-brown opacity
- ○ Post-eruptive enamel fracture

12. How often do you observe these lesions in the second temporary molar in comparison with the first permanent molar?

Tick one option

- ○ More often
- ○ Equally as often
- ○ Less often

13. Which factors do you believe are involved in the etiology of MIH?

Choose the corresponding answers

- ☐ Genetics
- ☐ Medicines taken by the pregnant mother
- ☐ Chronic medical condition that affects the pregnant mother
- ☐ Acute medical condition that affects the pregnant mother
- ☐ Fluoride
- ☐ Environmental pollutants
- ☐ Chronic medical condition that affects the child
- ☐ Acute medical conditions that affects the child
- ☐ Medicines taken by the child
- ☐ All the above
- ☐ Others
- ☐ Don´t know/No opinion

14. Do you believe the management of MIH is a challenge?

Tick one option

- ○ Yes, very difficult
- ○ Yes, somewhat difficult
- ○ No

15. If you answered yes to the previous question, where do you think the greatest difficulties lie?

Choose the corresponding answers

- ☐ Diagnosis
- ☐ Aesthetics
- ☐ Anesthesia management
- ☐ Correct determination of the margins of the tooth affected Providing correct restoration
- ☐ Long-term success of the restoration
- ☐ Behavior of the child
- ☐ All the above
- ☐ Other

16. Which factors influence the choice of restoration material in these cases?

Choose the corresponding answers

- ☐ Aesthetics
- ☐ Adhesion
- ☐ Preference of patient or parents
- ☐ Durability
- ☐ Potential for remineralization
- ☐ Sensitivity
- ☐ Personal experience
- ☐ Research results
- ☐ Others

17. Which materials would you use to restore these molars if they present post-eruptive fracture?

Choose the corresponding answers

- ☐ Pure glass ionomers
- ☐ Resin-reinforced ionomers
- ☐ Compomers
- ☐ Flowable composite
- ☐ Composite
- ☐ Stainless steel crowns
- ☐ Amalgams
- ☐ Inlays and onlays
- ☐ Silver diamine fluoride
- ☐ Others

18. Which materials would you use to restore these molars if they do NOT present post-eruptive fracture but only opacities?

Choose the corresponding answers

- ☐ Pure glass ionomers
- ☐ Resin-reinforced ionomers
- ☐ Compomers
- ☐ Flowable composite
- ☐ Composite
- ☐ Stainless steel crowns
- ☐ Amalgams
- ☐ Inlays and onlays
- ☐ Silver diamine fluoride
- ☐ Others

19. Which materials would you use to restore hypomineralized incisors?

Choose the corresponding answers

- ☐ Pure glass ionomers
- ☐ Resin-reinforced ionomers
- ☐ Compomers
- ☐ Flowable composite
- ☐ Composite
- ☐ Stainless steel crowns
- ☐ Amalgams
- ☐ Inlays and onlays
- ☐ Silver diamine fluoride
- ☐ Resin infiltrations
- ☐ Others

20. Are you receiving any information on MIH?

Tick one option

- ○ Yes
- ○ No

21. Where do you obtain the information?

Tick one option

- ○ Dental journals
- ○ Continuing education
- ○ Brochures
- ○ Internet
- ○ Books
- ○ Other

22. Would you like to receive more information on hypomineralization?

Tick one option

- ○ Yes
- ○ No

23. What do you need more information on?

Tick one option

- ○ Etiology
- ○ Diagnosis
- ○ Treatment
- ○ All

24. Clinical case. Seven-year-old patient with 3.6 semi-erupted and post-eruptive enamel fracture and sensitivity in the tooth. Which treatment option do you consider the best?

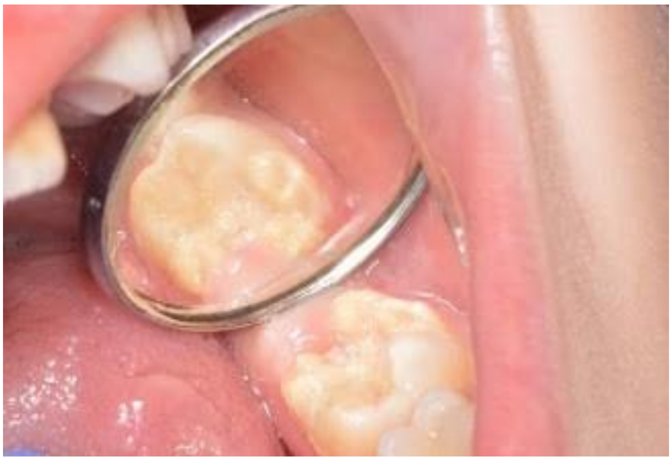

Tick one option

- ○ Fluoride varnish
- ○ Restoration with glass ionomer
- ○ Restoration with composite
- ○ Extraction
- ○ I am not sure of the best option

25. Clinical case. Which treatment option do you consider the best?

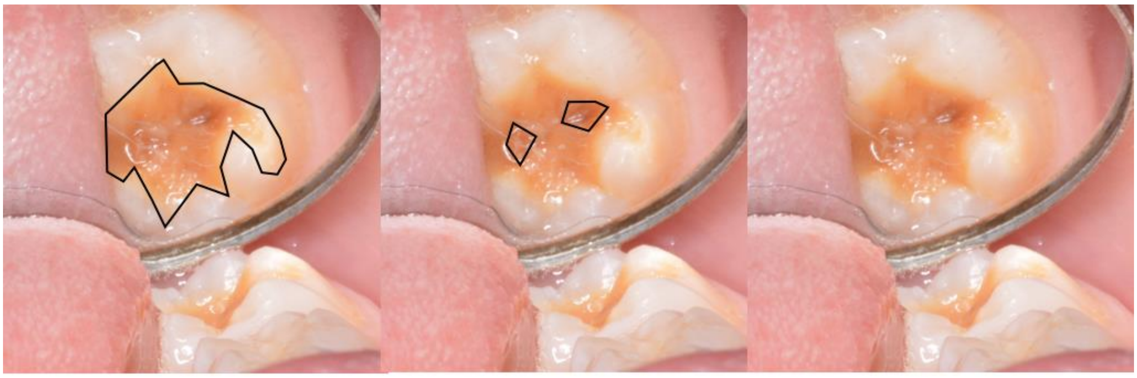

1.Eliminate all tissue affected by MIH until the healthy margin is reached	2. Eliminate only the most affected tissue	3. Do not eliminate any dental tissue
a. Composite restoration<br>b. Glass Ionomer Restoration<br>c. Temporary restoration	a. Composite restoration<br>b. Glass Ionomer Restoration<br>c. Temporary restoration	a. Composite restoration<br>b. Glass Ionomer Restoration<br>c. Temporary restoration

Choose the corresponding answers

- ☐ 1.a
- ☐ 1.b
- ☐ 1.c
- ☐ 2.a
- ☐ 2.b
- ☐ 2.c
- ☐ 3.a
- ☐ 3.b
- ☐ 3.c
